# Training SONAR spatial interpretation using virtual reality

**DOI:** 10.1186/s41235-025-00686-7

**Published:** 2025-11-05

**Authors:** John Salamon, Mike Nicholls, Irina Baetu, David Nicoll, Oren Griffiths

**Affiliations:** 1 College of Education, Psychology and Social Work, Flinders University, Adelaide, SA Australia; 2 School of Psychology, Faculty of Health and Medical Sciences, University of Adelaide, Adelaide, SA Australia; 3 School of Psychological Sciences, University of Newcastle, Callaghan, Newcastle, NSW 2308 Australia

**Keywords:** Spatial cognition, Decision aids, Virtual reality, SONAR, Automation

## Abstract

Submariners must interpret SONAR data and make rapid tactical decisions under challenging conditions. The broadband time-bearing plot is a common visualisation method which requires mental spatial transformations to generate an actionable representation. Participants (*N* = 81) were trained on a VR scenario that required them to spatially localize an enemy vessel indicated on a time-bearing plot. In the supported condition, participants were given access to a novel egocentric, conformal display, which wrapped the time-bearing plot around the user's field of view. The unsupported group received no assistance. Compared to a baseline unsupported condition, we found that the VR overlay markedly improved both speed and accuracy. Test performance revealed over-reliance in the supported group, but this phenomenon varied notably between participants. Those who jointly used the initial time-bearing plot and the conformal aid during training showed good performance with the aid, and no cost of prior exposure at test. Comprehension of SONAR data can be aided by novel display formats, but care must be taken to avoid over-reliance.

## Significance statement

Modern technical roles often require operators to make rapid interpretations of complex, high-level mathematical abstractions or plots of incoming sensor data. This study examines how novice adults learned to use time-bearing “waterfall” representations of broadband SONAR data—a common display format for submariners and other naval officers—in order to localize the depicted contact. We found that a novel, virtual reality-enabled display format markedly improved performance when present, but hindered subsequent unsupported performance. We found that over-reliance varied notably between people, with some people showing no cost of prior exposure to the support. These people were identifiable by their gaze distribution when the aid was present, which may inform policies regarding how visual supports are monitored and assessed during training.

## Training SONAR spatial interpretation using virtual reality

**Fig. 1:**
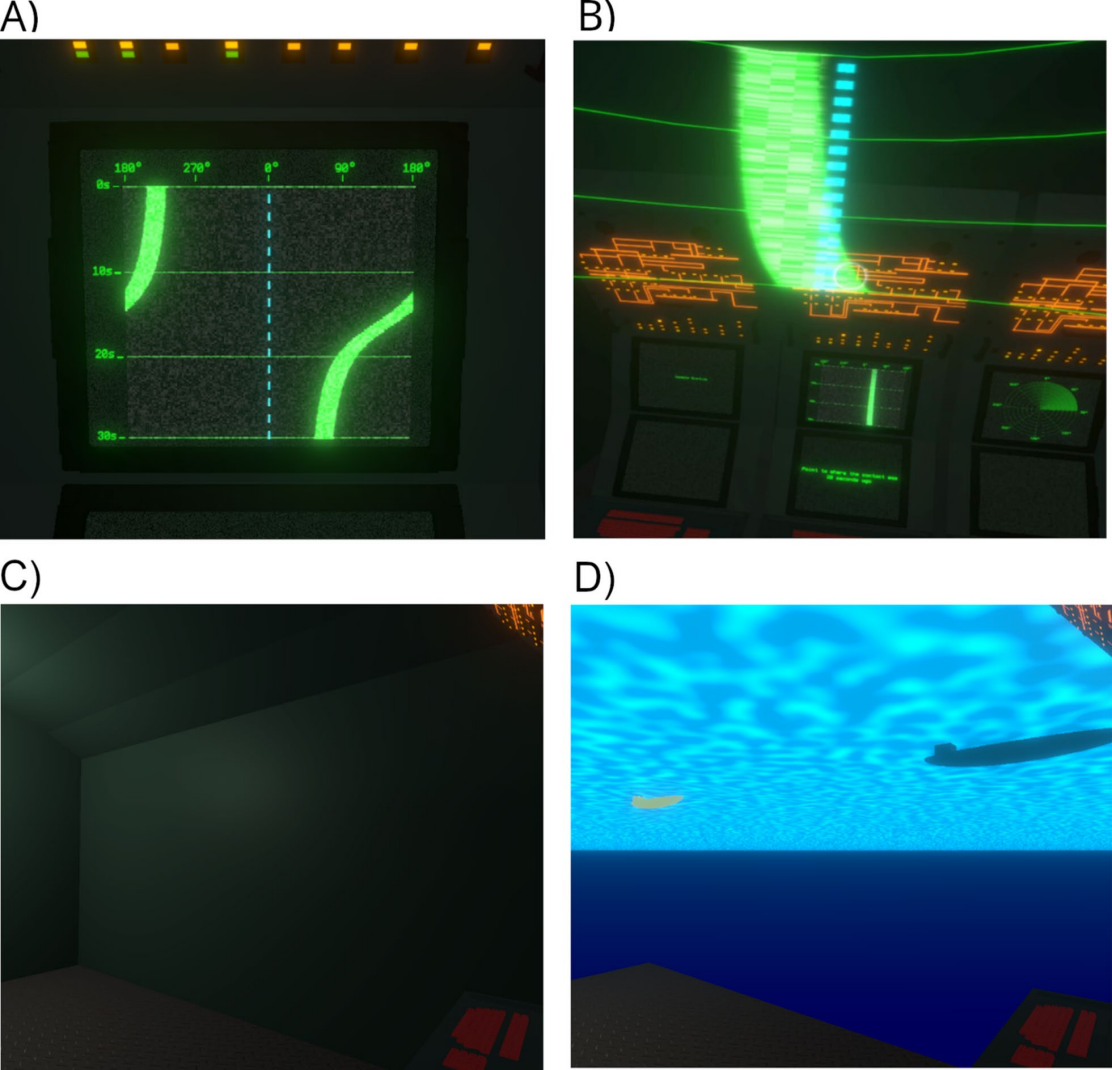
The virtual environment. *Note*: Screenshots taken of the virtual environment during the task. Panel **A** shows a sample time-bearing plot which appears on a fixed console in the VR scene. Plots of this kind were present continually for all phases and all participants. The foreground of Panel **B** shows a snippet of the virtual, semi-transparent overlay used to support decision-making, with the traditional time-bearing plot shown in a console in the background. This overlay was only offered to those in the Supported group. Panels **C** and **D** depict the participant’s perspective immediately before and after making an orienting response. Prior to the response, all the participant can see in the predicted direction is the submarine wall (Panel **C**). After indicating their response with a button click, the opaque wall fades to reveal the scene behind the wall (Panel **D**), thereby offering feedback. In this case, the participant has made an accurate response and the current position of the target vessel (grey) and its prior position (yellow) are both visible

In order to develop rich, spatial, situational awareness modern, SONAR operators on naval vessels need to perform transformations akin to *spatial perspective-taking*. That is, they must imagine themselves mentally occupying a different spatial position, including what can be “seen” from that position and its relative position. This skill—sometimes referred to as Type 2 perspective-taking (Flavell et al., 1986)—is needed to interpret broadband time-bearing SONAR displays. Such plots are also called Bearing Time Records or Waterfall Plots, and at first glance they resemble a conventional map. However, spatial information is reported as bearings (effectively a polar unit) organized in a Cartesian format whereby (x,y) pairings represent bearing and time (see Fig. 1a). Such plots are commonplace when navigating using passive SONAR, but place notable load on operators tasked with interpreting them (Fay et al., 2019). They require significant spatial skill to interpret as they require integrating information about the vessel upon which the viewer is boarded (commonly “ownship” in this literature); consideration of the effects of distance to the contact (not directly plotted); and they routinely involve non-linear mappings between what is depicted on the display and the velocity of the tracked contact (see Kirschenbaum et al., 2009, 2011, for detailed discussions). While there have been some efforts to simulate, gamify, or virtualize duties for the purposes of training or assessment (periscope operation, da Silva, 2016; officer on deck duties, Vincenzi et al., 2003), relatively little work has used virtual reality (VR) to explore whether novel display formats offer opportunities to improve SONAR operator skill acquisition and performance. The present experiment implemented a novel, VR device to present time-bearing plots in an egocentric, as distinct from allocentric, format and tested whether these plots would aid localization judgments in novice users, both when our novel support device was present and when it was subsequently removed.

### ***Time-bearing plots***

A broadband SONAR plot is primarily a two-dimensional display, with bearing plotted along the x-axis and time plotted along the y-axis (see Fig. 1a). The intensity of a signal (or contact) is often depicted in the intensity or colour of the icon shown at the intersection of the relevant time and bearing coordinates (thus ordinarily constitutes a third dimension, but was not used in our simplified task). This is the bright green, curved line shown in Fig. 1a, b. Typically bearing information is centred on North (0°) with the left and right extremes both indicating South (180°). Time is typically depicted with the most recent data appearing at the uppermost edge, with new information causing the older information to cascade down the display (akin to a waterfall). Operators use these plots in conjunction with additional information sources including raw acoustic information and other analytic methods and display formats, such as those used for Target Motion Analysis (or TMA), which more closely resemble the top-down allocentric display format used in conventional maps. The integration of data across these information sources is mentally taxing (Fay et al., 2019).

Consequently, many studies of cognition in submariners focus on higher-order challenges associated with data integration across roles. For example, researchers have explored the role of shared displays in facilitating distributed cognition amongst submariners (Michailovs et al., 2023, 2024); on visualization formats to help manage uncertainty during SONAR interpretation (Kirschenbaum et al., 2014); the effects of workload, interruptions and automated assistance on track management (Chen et al., 2017; Loft et al., 2015a, 2015b; Tatasciore et al., 2018). Less work has focused on the fundamental spatial skills necessary to build basic situational awareness using a time-bearing plot (see Hillyard et al., 1994).

Skilled navigators appear to effortlessly interpret these plots, but this ease is likely the product of substantial expertise (Kischenbaum et al., 2009). For example, a common judgment required of acoustic warfare operators is to identify the point of closest approach (Chen et al., 2017). Think for a moment about how that might that be represented on such a plot. Our intuition is that the answer to this question is not self-evident; the point of closest approach is indicated by an “S” pattern in the data, or more technically, where the rate of change in bearing is maximal. The present task centres on an even simpler, more foundational skill: mapping a single depicted contact to its location in egocentric space, or more straightforwardly, pointing to the indicated ship.

Making such judgments requires a surprisingly broad range of cognitive skills, and likely involves a complex interplay of discrete mental processes that commonly develop across childhood (Flavell et al., 1981), including mental rotation, inhibition, and spatial reasoning (Michelon & Zacks, 2006; Qureshi et al., 2010). Indeed, even if we limit our consideration to only those spatial reasoning skills underpinning a simple localization judgment, this is not a unitary domain. People vary widely both in spatial abilities (Wolbers & Hegarty, 2010) and in their preferred styles of spatial reasoning (Pazzaglia & De Beni, 2001). In a classic set of studies, Hegarty and Waller (2004, 2005) demonstrated that there are at least two dissociable skills evident in mental spatial transformations. The first, mental rotation, is conceptualized as performing a spatial transformation on an object within a fixed, egocentric spatial frame of reference. The second, perspective-taking, involves shifting one’s spatial frame of reference to be centred on that of a second object. Hegarty and Waller (2004) showed that these skills are distinct, such that performance on a range of tasks calling on these skills was best fitted by a two-factor solution (see Moraresku & Vlcek, 2020, for a review). While the correlation between the two factors was generally high (approximately 0.8), there is also convergent neural evidence for their functional dissociation (Zacks et al., 2003).

Both perspective-taking and mental rotation skills are called upon in the interpretation of time-bearing plots. Consider the simplest case, in which the orientation of one’s ownship (and indeed the officer’s own console) is static and oriented due north and the operator is only tasked with localizing a single contact with fixed velocity. Under Newcombe and colleagues’ influential taxonomy (Newcombe & Shipley, 2015; Uttal et al., 2013), this would likely constitute an intrinsic-dynamic task. That is, an operator is required to mentally locate an object in egocentric space, using a representation (the time-bearing plot) that largely aligns with their own (“up” = “in front”, “left” = “left” and so on). This task thus draws primarily on mental rotation skills, in that it requires calculating and monitoring the position (from incremental bearing information), of a second object (the contact), within a fixed or static and largely egocentric frame of reference. Within that frame, the tracked vessel will appear to incrementally rotate and translate.

However, the challenge faced by a SONAR operator is further complicated by two factors: (i) their ownship is likely heading in a direction other than due North, thereby bringing the ownship frame of reference into misalignment with the display; and (ii) the orientation of the console (and hence the display) may not be in alignment with the ownship direction of motion, leading to further systematic misalignment. These factors require drawing on perspective-taking skills, rather than pure mental rotation as it requires a change in the operator’s personal frame of reference. In Uttal et al’s (2013) framework, this would be an “extrinsic-dynamic” task. The scenario depicted in Fig. 1a is the simplest scenario, in which ownship heading (shown as the dotted blue line) is 0° and thus spatially aligns with the framework of the plot itself. Yet in our task, ownship heading was randomly selected per trial, so the dotted blue line could appear at any bearing. We anticipated that this factor, in combination with the movement of the target vessel, would challenge our participants. Spatial position appears to be often encoded relative to one’s own spatial position and orientation (Kessler & Thomson, 2010). Angular error increases with the deviation between the participant’s current facing direction and imagined orientation (Gardner et al., 2013).

While the translation of one’s own perspective is a cognitively challenging task, it is important to briefly note that these are far from the only complications a navigator must resolve. In realistic settings likely complications include: multiple contacts, notable signal noise, dynamic contacts, need to integrate SONAR data with other sources and/or analyses. Furthermore, the target may be taking active steps to disguise or cloak its signal, and there is no obligation for the contact (nor one’s ownship) to maintain a constant direction or speed; in fact, frequently changing heading is sometimes recommended as a means of disambiguating beamformed sensor information (Nardone & Aidala, 2007). These realities were simply too complicated for our novice learners. In order to keep our task accessible to novices, we allow ownship bearing to vary only between trials (not within a trial), and allowed the single depicted contact to be either (i) static or (ii) moving with a fixed velocity. We only showed a single contact per trial and did not include any false positives in the display. Because spatial skills vary notably between individuals, we further sought to investigate differences amongst individuals in pre-existing abilities (using a common, simple test of spatial ability; Peters et al., 1995). To anticipate, even this simplistic scenario proved challenging for most.

### A virtual, conformal, egocentric time-bearing plot

A core challenge of interpreting these displays is mapping the allocentric time-bearing plot to an egocentric frame of reference. A conceptually similar challenge was faced by aviators who previously sought to land their aircraft using displays not centred on their own perspective (Prevett, 1994), until perspective-oriented (or egocentric) displays were introduced and shown to be superior during final descent (Wickens & Prevett, 1995). However, on balance, perspectives not centred on the user (exocentric) retain some advantages over egocentric displays when the user’s goal is *understanding* a space (Thomas & Wickens, 1999), as distinct from navigating within it. These forms of navigation are likely separable skills, with differential brain activity patterns (van Asselen et al., 2006) and associated cognitive profiles (Sanders et al., 2008). Recent innovation with head mounted displays and optical see-through visualization methods have allowed novel integrations of egocentric and allocentric spatial representations, such as using virtual projections to “see through” walls (Xu et al., 2023). The present work continued that trend of dual format displays by examining whether semi-transparent overlays in VR of the (traditionally allocentric) time-bearing information could aid interpretation of traditional time-bearing plots in novices.

Specifically, we investigated the simplest direct translation of broadband SONAR to spatial knowledge as a proof-of-principle. All participants were first repeatedly shown a simulated SONAR broadband time-bearing plot depicting a single contact and they were asked to point in the direction of that contact in a VR scene. This was the “baseline” phase. In the second (or “training”) phase, half of the participants (the “supported” group) were provided with our additional display format. This overlay consisted of the same information contained in a standard time-bearing plot, but we used VR to wrap the time-bearing plot around the user’s head using a semi-transparent overlay, thereby converting the planar, allocentric display to a simultaneously presented egocentric, conformal display that was aligned with ownship heading. VR is particularly well-suited for this research question, because the display format is inherently egocentric; its primary benefit is its ability to foster immersion, which arises from its potential to put the user “in the scene” (with high extent of presence; Milgram & Kishino, 1994) and often assists spatial learning (see Abich et al., 2021, for a review). We anticipated that presenting the information in an egocentric format would reduce or remove the cognitive burden of translating the traditional time-bearing display into the egocentric mental representation needed to generate an orientating (pointing) response. We hypothesized this would be evident in faster and more accurate orienting responses when the VR overlay was present, relative to an otherwise matched control group (unsupported group) who completed the same training with only the traditional time-bearing plot. We anticipated the benefit would be particularly notable when the traditional display is substantially rotated from the participant’s own direction, thereby loading heavily on perspective-taking skills.

### Performance versus learning

Beyond the question of aiding performance, there is a second question pertaining to skill acquisition. VR has been used to both assess (He et al., 2022) and train or rehabilitate (Caglio et al., 2012; Faria et al., 2016; Masoumzadeh & Moussavi, 2020; Park, 2022) spatial cognition. Spatial skills, including perspective-taking, are highly malleable to training (Uttal et al., 2013), and may generalize beyond narrowly trained domains (Judd & Klingberg, 2021). Early studies demonstrated that training on a VR system built navigation skills in firefighters (Bliss et al., 1997), and naval officers (Hays & Vincenzi, 2000; Tate et al., 1997). There is now a large literature showing VR’s utility in training spatial abilities generally (Dunser et al., 2006; Abich et al., 2021). With respect to spatial perspective-taking skills, the Tangibles for Augmenting Spatial Cognition (TASC; Chang et al., 2017) system specifically improved spatial perspective-taking performance on standardised tests. In sum, focused training with VR supports can improve spatial perspective-taking skill, but we are unaware of any work examining our more narrow question: whether a VR egocentric display format would aid participants in learning how to interpret a time-bearing display.

Perhaps the more pertinent question is whether the present support allowed participants to experience a suitable level of difficulty to learn the underlying skill (i.e. place them in a “zone of proximal development,” Vygotsky & Cole, 1978). Providing conformal, egocentric information could, in principle, allow people to readily generate mental associations between an objects’ egocentric position and its onscreen position on the time-bearing plot. Mental associations of this kind generally form the fastest and most reliably when the to-be-associated stimuli are presented at the same time, and in tight spatial proximity (see Boakes & Costa, 2014, for a review). However, there are also costs to providing too assertive a support when people are initially learning a new skill; people need to experience “desirable difficulty” to acquire a new skill, and this can be hampered if assistance offered is sufficient to reduce the difficulty of the task below a critical level (Schmidt & Bjork, 1992; Wulf & Shea, 2002). This distinction is sometimes referred to as providing a “crutch” (whereby beneficial effects of the support are not retained when the support is removed) as distinct from a “scaffold” (whereby beneficial effects are retained with the support is removed).

Problematic reliance on a submariner decision support was observed in another study investigating interpretation of time-bearing plots albeit using a different tool in support of more complicated decisions (Chen et al., 2017). Consequently, we sought to design our VR overlay such that it would reduce the difficulty of the localization task, but not to the point that such judgments were trivial. In piloting, it appeared that challenging tasks (those with misalignments with current heading and those with a moving target) remained challenging for novices even with the display. While it appeared likely that the overlay would improve performance when it was present, clearly the more stringent test lay in whether this would translate into improved, maintained or impaired performance on a subsequent, unsupported test phase. Such over-reliance is not entirely determined by the tool, the decision and the environment. Individual difference factors play an important role (Szalma & Taylor, 2011). With this in mind, we closely inspected participants’ patterns of engagement with the VR overlay for hints of emerging over-reliance, by continuously monitored granular behavioural and eye-gaze metrics throughout the task. Exploratory gaze analyses were conducted to better understand participants’ attentional allocation during the task as optimal gaze behaviour has been associated with performance improvements in other complex navigation domains (in aeronautics; see Ziv, 2016, for a review).

## Methods

### Participants

Data collection began in October 2022 and was concluded in May 2023, during which eighty-one participants were recruited: 30 females and 51 males, with a mean age of 21.60 years (SD = 6.05). 65 were retained in the analyzed sample (see Results). Participants were asked brief questions about their experience with virtual reality games (one participant declined to answer). 37 participants reported that they played video games (on any platform) frequently, and 43 did not. The sample who noted that they played games declared an average of 7.13 h played in the past week (the average was 5.9 h per week including non-gamers). When asked what game they played most in the last week, 17 participants could not name a title, 11 reported games that were predominantly two-dimensional (The Sims, Snake, Candy Crush, Stardew Valley) and all others reported recent experience with titles that included three-dimensional navigation. Recruiting occurred at Flinders University, with the approval of the Flinders University Human Research Ethics Committee (#2023-5641). All participants reported normal or corrected-to-normal vision. Participants were compensated with either course credit or $20 for a single session lasting 1–1.5 h.

### Apparatus

The experiment was developed using the Unity game engine (version 2021.3.10f1) and the Unity Experiment Framework (Brookes et al., 2020). Participants viewed the experiment with an HTC Vive Pro Eye VR headset (90 Hz refresh, 1440 × 1600 pixel per-eye resolution, 110° field of view, 6° of freedom tracking). Eye and head tracking data were collected via the integrated sensors of the VR headset (120 Hz with 0.5–1.1° rated accuracy). Manual responses were collected using the pointing response and primary trigger buttons on the Vive “wands” (wireless pointing devices), synchronized to the headset. VR goggles were affixed to Wearable Sensing’s wireless DSI-24 dry electrode system which was used to simultaneously record EEG (19 scalp electrodes, 300 Hz). A single Vive controller was used to make directional responses.

### Virtual environment

Participants were placed in a simulation of a submarine control room, taking design cues from the size and design of the Australian Collins-class submarines (estimated from viewing a documentary commissioned by the national public broadcaster). The virtual room contained a row of three large consoles (consisting of two virtual screens each) directly in front of the participant and was otherwise empty. The virtual SONAR time-bearing plot was presented on the main screen of the centre console (Fig. 1a). When facing the SONAR console, participants were facing the forwards direction on the submarine. The virtual environment was set up in a play space of approximately 2.5 m × 4.5 m.

### Stimuli

In the simulation, the control room was placed below a virtual ocean, and a single external contact (i.e., indicated by a 3D ship model at the surface of the ocean) was placed at an initially random location on the perimeter of a horizontal 20 m circle around the control room. The submarine control room was located at periscope depth (that is, the in-game room scene was situated just below the water line). The ship model representing the target contact was coloured grey, and a second copy of that ship model was also shown. This copy of the target was colour-shifted to appear yellow and was presented at the location the target contact occupied 20 s prior to the participants’ response time (to provide feedback for their second judgment, see Procedure). For trials in which the target held a fixed location, the two ship models were presented overlaid on each other.

The control room scene was arranged to facilitate the fading of the room’s walls at the moment the participant registered their location response. When that occurred, the walls faded away and participants could visually observe the actual location of the contact (the grey ship model) and its previous location (the yellow ship) during training periods in which feedback was offered. The time-bearing plot and overlay remained visible while the submarine walls were hidden. In addition to the two ships representing the contact’s true locations (at time of decision and 20 s prior), two icons were shown to indicate the directions that participants had just guessed. One grey cube indicated whether they guessed the contact was currently, and a second yellow cube (coloured yellow) indicated where they estimated it was 20 s prior. Participants could see an in-scene projection of the current position of the wand controller, and the direction the controller was indicated using a green ray cast in the direction it was currently oriented.

With respect to the time-bearing plot (and the VR conformal overlaid display), the actual location of the contact was updated on the SONAR screen at a rate of 4 Hz and was represented by a luminous green line on the constantly updating time-bearing plot (as seen in Fig. 1a, b). A blue dashed line on the display indicated the current direction of motion of the submarine. The y-axis of the plot began at zero seconds (current time) at the top and extended 30 s down to the lower edge. Horizontal tick markers indicated every 10 s. On the x-axis, tick markers were shown for each cardinal direction, along with a numeric indicator of the corresponding angle in degrees (0°, 90°, 180°, 270°). Full source code is available at OSF (see *Data Availability*).

### Procedure

Participants gave informed consent and were then administered a pen-and-paper mental rotation test before beginning the VR task. Specifically, they completed Section A of the 24-item form of the Revised Vandenburg & Kuse Mental Rotation Test (Peters et al., 1995). This was administered individually in a quiet room and was untimed. Participants were then instructed using a verbal script (available at OSF; see *Data Availability*). These instructions were supported by a visual aid displaying an image of the in-game scene, and an image of a time-bearing plot similar to those shown in Fig. 1. The visual examples were used to explain how to interpret our simulated SONAR time-bearing plots by explaining each axis and giving three demonstrations questions to check for understanding (for example, if a ship was shown here on the display, please point to where that ship would be relative to you?). This process took 5–10 min, depending on the number of clarifying questions asked. Only when this was complete was the headset applied. Participants were then randomly assigned to either the supported (*N* = 41) or unsupported (*N* = 40) condition, and the experiment commenced.

Participants were then fitted into the DSI-24 EEG headset, and a pair of HTC Vive VR goggles. Once the simulation commenced, people were acclimatized to the virtual control room. They were asked to move around the space and find a standing position that was a comfortable distance in front of the primary SONAR console.

The simulation task was divided into three phases: Baseline, training, and test. All phases were 40 trials in duration (although the baseline phase included an additional 10 practice trials, described below). In each phase, participants were presented with different configurations of submarine heading, contact position and contact speed on every trial. Each trial commenced with the participant staring at the console for approximately 3 s. The trial only commenced when they were oriented to the console (equivalent to “looking forwards” in our subsequent analyses). The time-bearing plot would then populate with a single contact (Fig. 1a). Half of the trials were static, in which the target held a constant bearing throughout the trial. On these trials, the time-bearing plot resembled a vertical line (i.e., a fixed bearing varying only across time). Half of the trials were dynamic, in which the target moved with a fixed velocity. These typically generated a wave like pattern on the time-bearing plot, as shown in Fig. 1a. The heading of one’s ownship was indicated using a dotted blue line and was randomly chosen on each trial. Ownship heading, initial contact position and contact velocity were free to vary independently of each other on every trial. There were two exceptions to these rules during the initial 10 practice trials. Practice trials started with easier scenarios in which the first three trials had the ownship heading fixed at 0° and the target vessel was stationary. Over the ten practice trials, these constraints were gradually relaxed such that by the tenth trial the contacts on each trial were dynamic and ownship heading was randomly selected. During the practice trials, participants were allowed to ask for assistance or clarification, but in practice this did not often occur.

Participants were asked to make two judgments per trial. We asked them to point first to where the contact was 20 s ago and register their first response (by clicking a button on a handheld wand synchronized to the HTC headset). Then they needed to move the wand to where they believed the contact was currently and press a second button on the wand. The judgment of where the vessel was 20 s ago was facilitated by a tick mark and gridline at 20 s on the vertical axis of the time-bearing plot (and equivalently on the virtual overlay). Both responses needed to be registered within 15 s. Two trials were required to ascertain participants’ ability to interpret both axes (time and bearing).

**Fig. 2:**
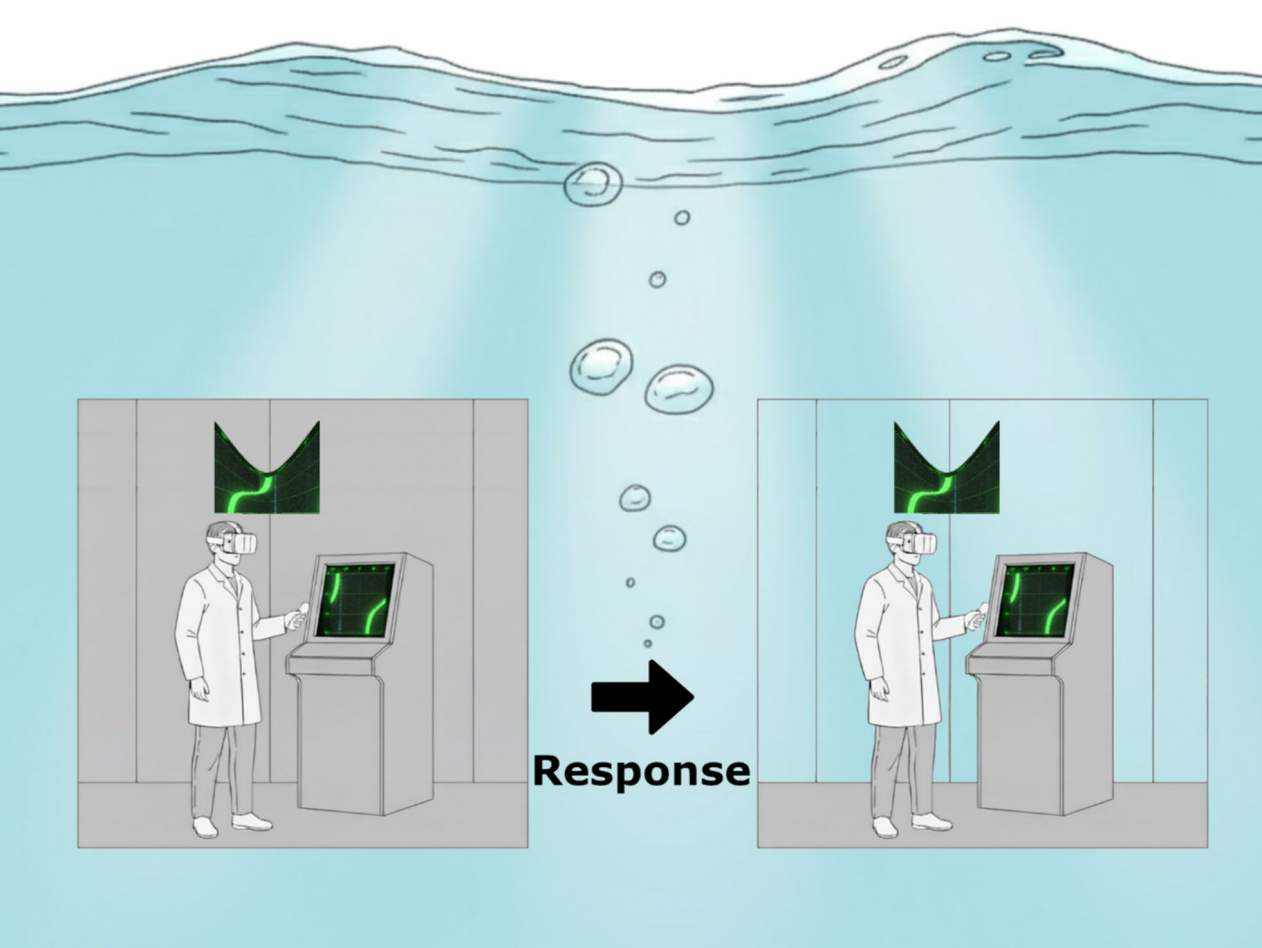
Procedure. *Note*: Schematic of the procedure within each trial during the training phase for the supported group. Participants commenced each trial in a virtual grey room with opaque grey walls, and with a time-bearing plot shown on the console. For participants in the supported group during the training phase, there was an additional display visible as a semi-transparent overlay (this was not present during training or test phases for the supported group, and was never present in the unsupported group). This is shown as the curved display hovering overhead. In the virtual, conformal image the same time-bearing plot is reoriented to the user’s perspective and wrapped around their visual field, just above the eyeline. On all other phases, participants only had the time-bearing plot on the console available. Once a response was made, the walls became transparent (as shown in the right-hand image) and the participant can determine if they made a correct response or not by looking for the grey/yellow ship icons (see Fig. 1D). Feedback was not provided in the final, test phase for either group

Once both judgments were registered, feedback was provided in the baseline and training phases (not the test phase). During the feedback period, the walls of the submarine would fade out (see Fig. 2). This would reveal the actual location of the target (represented as a grey ship) icon and its previous location (a yellow ship icon), as well as two floating cubes. The two floating cubes represented the participants’ two bearing judgments for the present and prior positions of the ship (as grey and yellow cubes, respectively). During static trials, the grey and yellow ship icons were in the same position (and only a single grey ship was visible). When a participant guessed correctly, the cube icons overlay the ship icons, but both were visible. After registering a response, participants had 4 s to view their feedback, before needing to return their gaze to the forward console so as to signal readiness to commence the next trial.

Training proceeded in this manner across all three phases for the unsupported group, with the only difference across phases being that feedback was withheld during the final test phase. For the supported group, during the second (training) phase a semi-transparent overlay was shown on each trial (see Fig. 1b). This semi-transparent, conformal display represented the time-bearing plot translated to reflect the direction of the ship’s own heading and wrapped around the participants’ head. This meant that bearing information shown in the traditional time-bearing plot was now rendered egocentric, not allocentric. This display was present during the training phase only and was removed for test. In all other respects, the two groups were treated identically.

Gaze data and electrophysiological responses were measured continuously throughout the task. So as to focus our analyses on the decision period, gaze data reported herein were collected continuously between the onset of the trial and the completion of the participants’ response. Electrophysiological responses had excessive motion artifacts due to the speeded, pivoting, twisting motion inherent in the task as a result they are not presented here. The following analyses are limited to behavioural and gaze measures.

### Analysis

Accuracy was quantified as a continuous measure of degrees from correct target (1.0 = 0° difference, 0.0 = 180° difference). Response latencies were measured from the initial presentation of data on the SONAR screen. Accuracy and latency for the first and second orienting responses per trial were very highly correlated (*r* > 0.8), so for all analyses the accuracies and latencies across the first and second response were averaged to obtain mean accuracy and latency per participant per trial.

Two additional measures were used to interpret hand position and gaze data. The first quantified the time spent looking forward. This “look forward ratio” measured the time on each trial the participant’s eye position was within 30 horizontal degrees (in either direction) of the SONAR time-bearing plot. The second quantified the time spent looking at the target contact (with the same margin); the look target ratio. The two windows were not exclusive: if the target appeared in a forward position on a particular trial, the two gaze measurement windows (look target and look forward) could overlap. Because trial duration varied between and within subjects (as the trial terminated upon response), both metrics were normalized by trial length and thus expressed as a proportion of each trial or “ratio.”

## Results

All analyses controlled the Type I error rate at 0.05 using Bonferroni corrections where possible. Frequentist statistics were used for inferential purposes (primary *z*, *t*, *F* and *χ*^*2*^ tests). Bayes Factors were reported as evidence favouring the alternative hypothesis (“BF10") and were interpreted according to Jeffreys (1961). All analyses were conducted using JASP (2024) using default settings. For linear mixed models, we used Satterthwaite estimation of degrees of freedom. Effect size estimation was conducted using standard measures (*η*_*P*_^*2*^, Fisher’s z, Cohen’s *d*) or confidence intervals, wherever possible.

### Determining learners

**Fig. 3:**
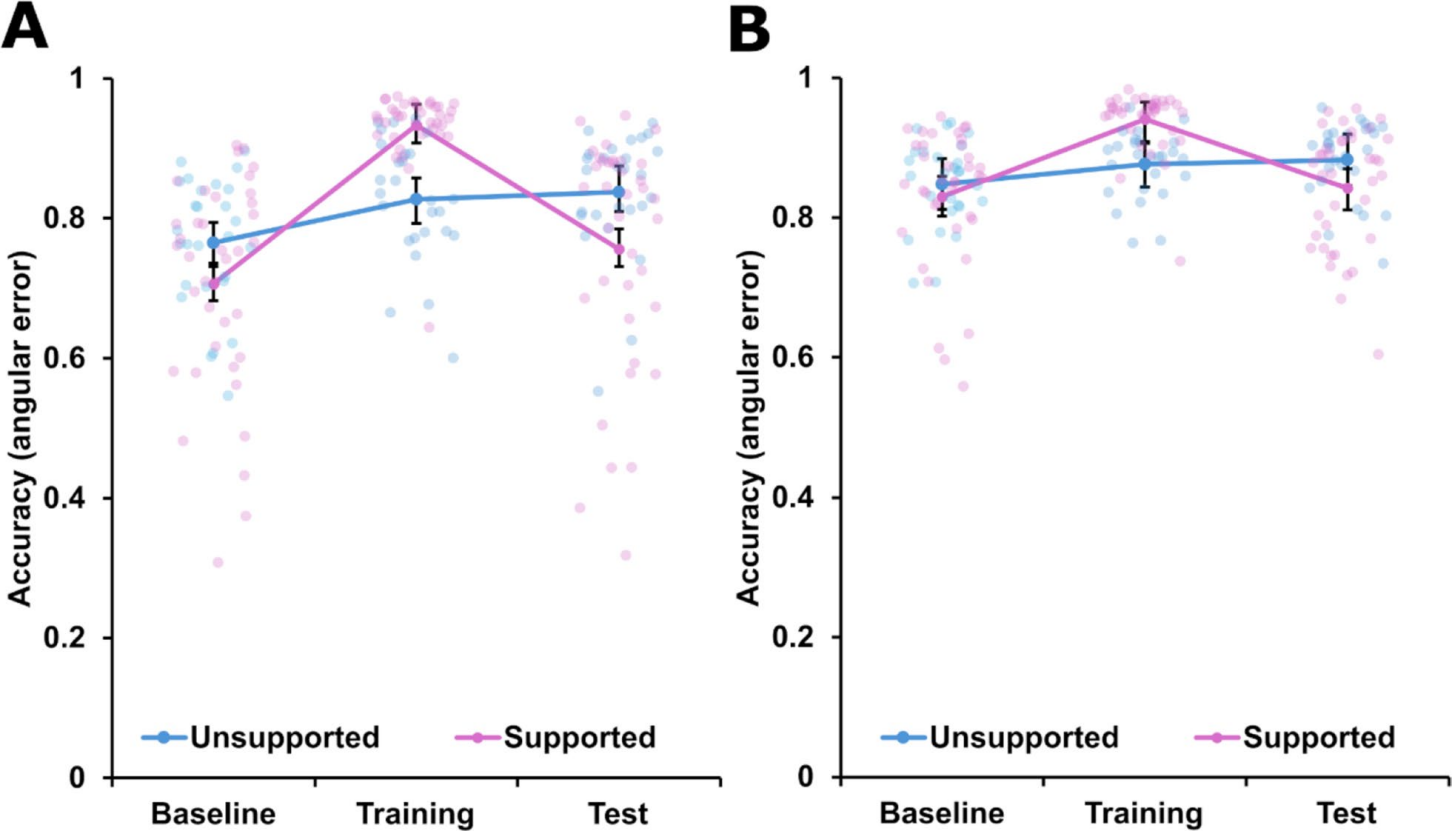
Pointing accuracy by phase and group. *Note*: Mean accuracy of participants across the experiment, separated by trial-type. Accuracy was quantified as a continuous measure of degrees from correct target (1.0 = 0° difference, 0.0 = 180° difference). Panel **A** shows trials in which ownship heading and participant’s orientation were misaligned (non-forward-facing) while the right-hand panel **B** shows trials in which they were aligned (forward-facing; see Results for detailed calculations). The y-axis indexes accuracy whereby 1 equals a pointing response that deviated less than 1° from the target vessel, and a 0 indicates a point error of 180°. The solid lines and filled icons represent group means in each figure, and the vertical error bars indicate 95% confidence intervals. The semi-transparent circles at each data point represent the mean performance of one individual participant

Our primary interest lay in the impact of the conformal visual support on people’s ability to perform the localization task, and on their ability to continue to perform that task when the display was removed. To examine our first question, we scored each trial as correct or incorrect (binarization was used to facilitate comparing against chance; all subsequent analyses were performed on continuous accuracy data). Each response was coded as correct if the estimated current target location was within 30 horizontal degrees of its true location, and incorrect otherwise. We accepted a broad of values as correct (30°) under this analysis so as to be conservative with respect to exclusions. Participants who had some idea of where the target was but who may have been inaccurate in their representation or execution would not meet the exclusion criteria and would thus be included in our analyses. A binomial test (against a 50% accuracy baseline) was then performed for each individual participant to examine if their location responses exceeded chance. 65 of 81 participants exceeded chance during the training period, and this distribution was impacted by the supported condition: 38/41 supported participants exceeded chance, while only 27/40 unsupported participants exceeded chance, *χ*^*2*^(1) = 8.10, *p* = 0.004. A similar calculation was performed to examine whether self-reported regular gaming experience had any impact on the likelihood of being classed as a learner. Prior gaming experience had no detectable impact: 29 of 37 people who played games regularly exceeded chance performance on our task, as compared with 35 of 43 non-gamers, *χ*^*2*^(1) = 0.01, *p* = 0.91. In summary, our novel support allowed more novice participants to complete the task than those not offered the support, irrespective of their prior experience with video games. All subsequent analyses were performed on the subset of learners (38 supported, 27 unsupported) who were able to demonstrate some above-chance capacity for the task during the second, training phase. Mean performance of these participants across all phases are shown in Fig. 3.

### Pointing accuracy and latencies

Mean performance for both groups (supported and unsupported) across each phase (baseline, training and test) are shown in Fig. 3. Trial-level data were entered into a single linear mixed model with four fixed factors: phase (baseline, training and test), group (supported, unsupported), target speed (fixed, moving) and heading (forward, else). A random-effect intercept was also entered for participant identity. For heading, we separated trials based on ownship heading. If ownship was within 30° of North, then the participants’ perspective roughly aligned with the time-bearing plot (similar to how the “look forward” ratio was calculated). These “forward-facing” trials were expected to be less demanding than the other trials (labelled “non-forward-facing”). The participant random-effect allowed us to model the inter-individual differences in pre-existing ability and performance.

There were significant main effects of phase *F*(2, 8122.34) = 196.99, *p* < 0.001, *η*_*P*_^*2*^ = 0.05, heading, *F*(1, 8126.26) = 197.62, *p* < 0.001, *η*_*P*_^*2*^ = 0.02, and boat speed, *F*(1, 8120.88) = 103.65, *p* < 0.001, *η*_*P*_^*2*^ = 0.01. The main effect of group was not significant, *F*(1, 63.68) = 0.05, *p* = 0.82, *η*_*P*_^*2*^ < 0.001. As shown in Fig. 3, participants were generally more accurate when they were facing forwards, and both groups tended to perform more accurately in the latter two phases (training and test) than in the baseline phase. Participants were generally more accurate when the target was static than dynamic (comparison not shown).

The effects of primary interest were those involving the interaction of phase and group. Phase significantly interacted with group, *F*(2,8122.34) = 95.46, *p* < 0.001, *η*_*P*_^*2*^ = 0.02. The effect of phase on differences between the groups was not involved in a three-way interaction with boat speed, *F*(2, 8120.87) = 0.92, *p* = 0.41, *η*_*P*_^*2*^ < 0.001, but it did significantly interact with heading, *F*(2, 8125.99) = 11.72, *p* < 0.001, *η*_*P*_^*2*^ = 0.002. As can be seen in Fig. 3, the same general pattern held across both headings (shown individually in the left and right panels), but the differences between groups were more evident in the non-forward-facing trials (left panel).

To unpack this interaction, simple effect contrasts examined the impact of group membership (supported, unsupported) on performance during each phase when considered individually. We further divided these comparisons by heading, for a total of six simple comparisons. These were performed using Bonferroni-corrected, 95% confidence intervals with asymptotic *z*-tests (under JASP’s default settings, i.e. degrees of freedom were large and not estimated). During the baseline phase, prior to introduction of the support, no significant difference between groups was observed on either forward-facing or other trials: the mean accuracy difference between the groups on forward-facing trials was 0.02 (equivalent to 4° difference), 95% CI [− 0.03, 0.06], *z* = 0.69, *p* = 0.99, and on other trials it was 0.05, (or 9°), 95% CI [0.01, 0.10], *z* = 2.53, *p* = 0.07. Interpretation of the latter finding was complicated by the fact that the confidence interval did not cross 0 (suggesting a significant effect), but with an appropriate statistical correction it did not meet the 0.05 criterion. To help disambiguate this conclusion, a simple t test was conducted between the groups (supported versus unsupported) on the non-forward-facing trials in the baseline phase. The effect was not significant, with the Bayes Factor suggesting the comparison provides no evidence for a difference nor for its absence, *t*(63) = 1.81, *p* = 0.08, *d* = 0.26, *BF* = 1.00.

During the training phase when the support was present, the supported group was significantly more accurate than the unsupported group on non-forward trials by 0.11 (or 20°), 95% CI [0.07, 0.15], *z* = 5.13, *p* < 0.001, but not on forward-facing trials (mean difference = 0.06 or 10.8°), 95% CI [0.01, 0.10], *z* = 2.45, *p* = 0.09. In the test phase, when the supports were removed, the unsupported group were more accurate on non-forward-facing trials, by 0.08 (14.4°), 95% CI [0.04, 0.13], *z* = 3.92, *p* < 0.001, but not on forward-facing trials (0.04, 7.4°), 95% CI [− 0.01, 0.09], *z* = 1.76, *p* = 0.48. In summary, the supported group was more accurate than the unsupported group when the overlays were present, but the pattern reversed when those overlays were removed. These differences were most evident when ownship heading and the time-bearing plot were misaligned.

The same structured analysis was used to analyse response latencies. Mean response latency by group and phase are shown in the upper panel of Fig. 3. In the generalized linear mixed model analysis, all four main effects were significant: phase, *F*(2,8121.78) = 572.63, *p* < 0.001, *η*_*P*_^*2*^ = 0.12; group, *F*(1,63.40) = 6.55, *p* = 0.01, *η*_*P*_^*2*^ = 0.09; heading,* F*(1, 8125.00) = 58.05, *p* < 0.001, *η*_*P*_^*2*^ = 0.007; and boat speed, *F*(1, 8120.57) = 409.70, *p* < 0.001, *η*_*P*_^*2*^ = 0.05. Overall, the participants in the supported group responded faster (than the unsupported group), participants increased response speed across phases (they were the slowest in the initial baseline phase), and people responded faster when the target was stationary and their orientation was closely aligned with ownship heading.

Again, our primary interest lay in the interactions between phase and group. The two-way interaction between phase and group was significant, *F*(2, 8121.78) = 8.18, *p* < 0.001, *η*_*P*_^*2*^ = 0.002, and this was not significantly moderated by a three-way interaction with boat speed nor heading: boat speed, *F*(2, 8120.56) = 0.42, *p* = 0.66, *η*_*P*_^*2*^ < 0.001, and heading, *F*(2, 8124.01) = 0.33, *p* = 0.72, *η*_*P*_^*2*^ < 0.001. The interaction between phase and group was decomposed into simple effects examining the effect of group during each phase, when considered in isolation. Bonferroni-corrected z tests were used to analyse contrast estimates. During the baseline phase, no significant difference between groups was observed: the mean difference in response latency was 0.65 s (in favour of the supported group), 95% CI [0.06, 1.30], *z* = 1.98, *p* = 0.14. During the training phase, when the supported was present, the supported group responded 1.20 s faster than the unsupported group on average, 95% CI [0.54, 1.85], *z* = 3.58, *p* < 0.001. During the final test phase, when the supports were removed, the supported group responded 0.60 s faster on average, but this was not a significant difference, 95% CI [− 0.05, 1.26], *z* = 1.81, *p* = 0.21. In summary, the supported group generally responded faster than the unsupported group, but this difference was largely contained in the training phase when the support was present (a small numeric difference during the baseline phase did not survive statistical correction for multiple comparisons).

### Hand and eye tracking measures

Exploratory eye tracking analyses were conducted. Each gaze metric was inferentially analysed in the same manner as behavioural response accuracy and latency. We hypothesized that whenever participants were not offered the visual overlay they would be forced to spend more time looking at the time-bearing plot in order to localize the target. The raw proportions observed for look forward ratios are not themselves meaningful. People were required to return gaze to the centre console at the end of each trial in order to trigger the next, so it is likely that any individual ratio is an overestimate of the time needed to examine the time-bearing plot. Nevertheless, the change in this ratio across trials and conditions is instructive as the requirement to return gaze to the console between trials was fixed across all phases and conditions.

**Fig. 4:**
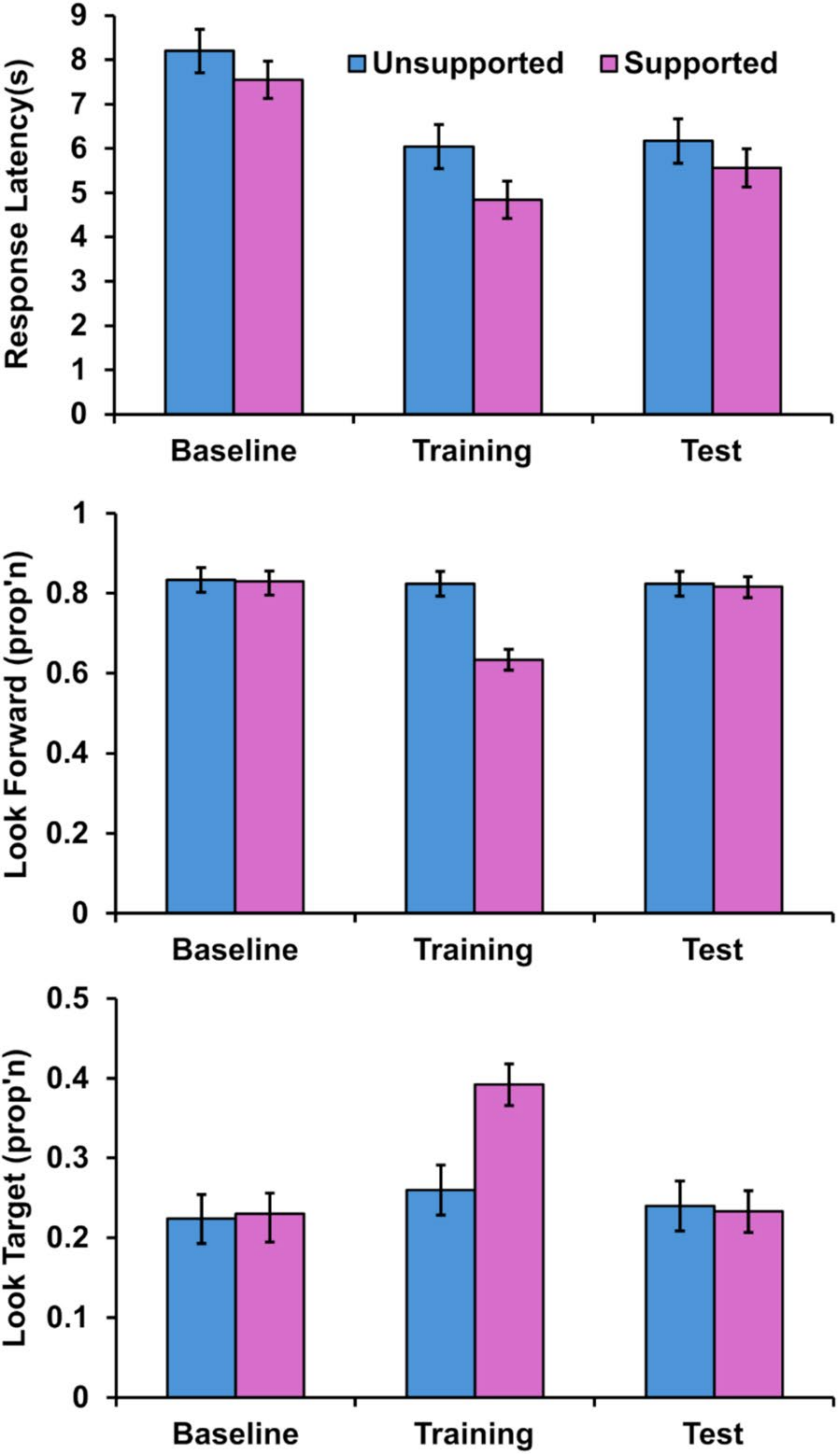
Response latency and gaze ratios. *Note*: All panels show group mean performance divided by phase (baseline, training, test) and group (unsupported, supported). The upper panel shows mean response latencies for pointing responses (seconds). The middle panel shows the mean proportion of decision time participants spent looking in the direction of the traditional time-bearing plot (look forward ratio). The lower panel shows the mean proportion of time people spent gazing in the direction of the target prior to responding. Error bars represent 95% confidence intervals in all panels

All main effects were significant: phase, *F*(2, 8335.18) = 299.15, *p* < 0.001, *η*_*P*_^*2*^ = 0.07; group, *F*(1, 63.53) = 11.45, *p* < 0.001, *η*_*P*_^*2*^ = 0.15; heading, *F*(1, 8338.42) = 17.63, *p* < 0.001, *η*_*P*_^*2*^ = 0.002; and boat speed, *F*(1, 8334.40) = 12.40, *p* < 0.001, *η*_*P*_^*2*^ = 0.002. People looked forward slightly more when the boat was moving (static *M* = 0.79 versus dynamic *M* = 0.80), when they were not facing forward (forward *M* = 0.79 versus else *M* = 0.80), if they were in the unsupported group and in the two phases in which the overlay was absent. As can be seen in Fig. 4, those latter two main effects are primarily due to people looking forward much less when the overlay is present.

This interpretation was supported by the significant interaction between phase and group, *F*(2, 8335.18) = 261.50, *p* < 0.001, *η*_*P*_^*2*^ = 0.06. This two-way interaction was further moderated by boat speed, *F*(2, 8334.63) = 12.93, *p* < 0.001, *η*_*P*_^*2*^ = 0.03, but not heading, *F*(2, 8337.40) = 0.75, *p* = 0.47, *η*_*P*_^*2*^ < 0.001. To unpack these interactions, we again looked at the simple effect of group in each phase separately, but we additionally tested the effect of boat speed on looking forward ratio in the supported group during the training phase. During the baseline and test phases, no significant differences between groups were observed: baseline, 95% CI [− 0.07, 0.09], *z* = 0.21, *p* = 1.00 and test, 95% CI [− 0.07, 0.10], *z* = 0.39, *p* = 1.00. Participants in the unsupported group looked forward more than those in the unsupported group in the training phase, 95% CI [0.30, 0.46], *z* = 9.18, *p* < 0.001. When the supported group was examined alone in the training phase, participants looked forward more during the static trials (*M* = 0.66) than the dynamic trials (*M* = 0.61), 95% CI [0.04, 0.07], *z* = 6.09, *p* < 0.001. In summary, people looked forward at a consistent rate on each trial (approximately 0.7 to 0.8 of the gaze time) except when the overlay was present (dropped to around 0.6). People generally tended to look forwards on dynamic trials, suggesting a close inspection of the challenging time-bearing plot in order to locate the moving target. However, when the overlay was present, the opposite pattern was observed: on average, people spent less time looking forward on dynamic trials.

Turning to the other gaze metric, the look target ratio, we see a very similar pattern. People tended to look at the target at a very similar ratio across all trials when the overlay was absent (approximately 0.25), but in the one phase/condition where the overlay was present, this value increases by approximately 0.40. This is unsurprising because the target direction is typically an unremarkable patch of the submarine internal wall when the overlay is absent; it is only once a response is generated that the wall disappears and the target vessel is revealed. When the overlay is present, there is a semi-transparent green line at that target’s visual location. These ratios were inferentially analyzed in the same manner. There were significant main effects of phase, *F*(2,8342.62) = 75.21, *p* < 0.001, *η*_*P*_^*2*^ = 0.02; group, *F*(1,67.37) = 10.20, *p* = 0.002, *η*_*P*_^*2*^ = 0.13; heading, *F*(1,8365.67) = 22.76, *p* < 0.001, *η*_*P*_^*2*^ = 0.002; and boat speed, *F*(1,8337.24) = 36.69, *p* < 0.001, *η*_*P*_^*2*^ = 0.004.

Phase significantly interacted with group, *F*(2, 8342.62) = 35.92, *p* < 0.001, *η*_*P*_^*2*^ = 0.008, but there was no significant three-way interaction with boat speed, *F*(2, 8338.99) = 1.02, *p* = 0.36, *η*_*P*_^*2*^ < 0.001, nor heading, *F*(2, 8359.21) = 0.07, *p* = 0.94, *η*_*P*_^*2*^ < 0.001. As previously, three simple effect contrasts examined the effect of group within each phase. The two groups did not differ in their look target ratio during baseline, 95% CI [− 0.04, 0.03], *z* = 0.37, *p* = 1.00, nor in the test phase, 95% CI [− 0.03, 0.04], *z* = 0.42, *p* = 1.00. During the training phase, the supported group looked towards the target for longer during the decision period, 95% CI [0.10, 0.17], *z* = 7.58, *p* < 0.001.

### Individual differences

**Fig. 5:**
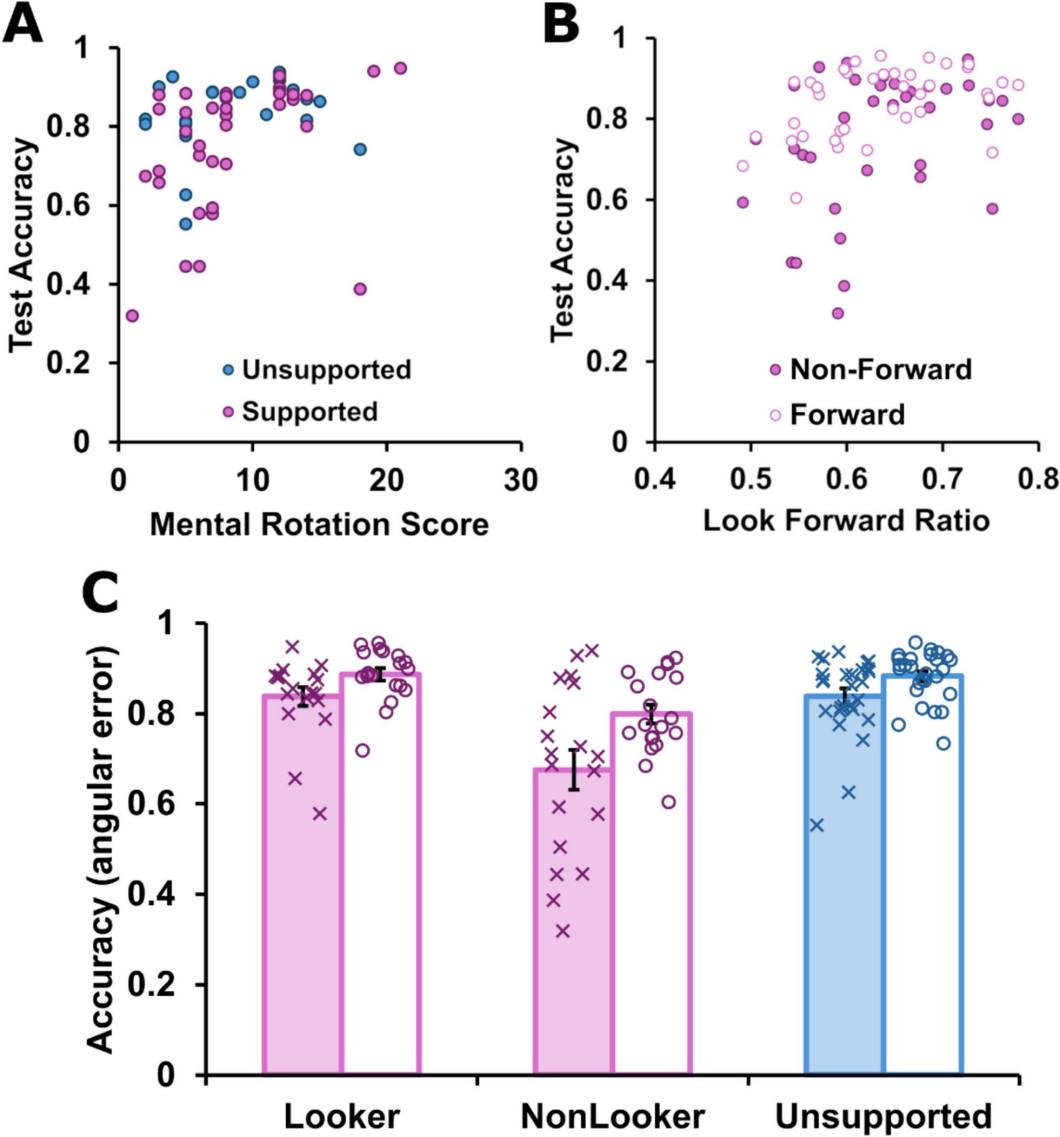
Individual differences in spatial ability, gaze and test accuracy. *Note*: Relationships between mental rotation ability, look forward ratio and response accuracy at test. In all panels, purple icons and columns refer to data from the supported group, and blue refers to data from the unsupported group. Panel **A** shows the correlation between total scores on the mental rotation test and accuracy on the difficult, non-forward-facing trials at test. Each point represents a person’s mean performance, with colour indicating group membership. Panel **B** shows the relationship between looking forward during the training phase (look forward ratio) and accuracy on the two trial-types at test (forward-facing and non-forward-facing). Note that each participant in the supported group is represented by two points in this image. Panel **C** shows mean performance during test, divided by trial type (forward-facing in open columns and non-forwarding-facing in filled columns). The supported group is further divided into “lookers” and “non-lookers” based on the degree to which they looked forward during the training phase. Error bars represent standard error of the mean. The mean performance of individual participants is shown with overlaid “X” and “O” icons

There are notable individual differences in both spatial ability and how people tend to interact with automation (Hegarty & Waller, 2004). To this end, we sought to test focused hypotheses concerning how these two types of individual differences impacted on performance. First, we considered pre-existing general spatial ability, tested with a pen-and-paper mental rotation task (Peters, 1995). A simple bivariate correlation was calculated between performance on the pen-and-paper mental rotation task, and mean accuracy at test on the non-forward-facing trials that requires a perspective shift (those in which the participant’s orientation was misaligned with ownship heading). Overall, mental rotation skill significantly predicted accuracy at test, *r*(63) = 0.32, *p* = 0.01, *z* = 0.33 (see Fig. 5a). If the visual overlay was sufficiently supportive that it reduced or removed any need for pre-existing spatial ability, then we would expect there to be no such relationship between mental rotation skill and accuracy when the support was present. Consistent with this prediction, the correlation between mental rotation scores of people in the supported group, and their accuracy on the difficult, non-forward-facing trials during the training phase, was not significant: *r*(36) = 0.20, *p* = 0.23, *z* = 0.21.

A second analysis concerned the participants’ engagement with the time-bearing plot. In operationalizing what might discriminate people who were over-reliant on the conformal display cue as distinct from using that cue to facilitate spatial learning, we anticipated that those who became reliant would essentially ignore the time-bearing plot and instead rely wholly on the conformal cue. In contrast, those who used the conformal cue to teach themselves how to interpret the time-bearing display might alternate between viewing the conformal display cues and the time-bearing plot in the course of generating their location response. Such behavioural differences ought to be evident in differential scores on our look forward ratio in those given the support: those who ignored the time-bearing plot would have lower scores, and those who use the time-bearing plot in conjunction with the aid would have higher scores. We found that the look forward ratio of supported participants on the non-forward-facing trials during training (when the conformal display was present) predicted later accuracy on both non-forward-facing trials, *r*(37) = 0.35, *p* = 0.03, *z* = 0.37, and on forward-facing trials, *r*(37) = 0.44, *p* = 0.005, *z* = 0.48.

Given the evidence of over-reliance in our group level analyses, we further examined whether all participants in the supported group demonstrated such over-reliance at test. Specifically, we performed a median split on the supported group based on their look forward ratio during training (i.e. the degree to which they sought to use the conformal cues for learning as well as performance), and compared the two subsets of the supported group (“lookers” and “non-lookers”) against the unsupported group at test. Consistent with our primary analyses, we examined performance separately on the forward-facing and non-forward-facing trials at test, resulting in a 3 × 2 mixed factors ANOVA, with a between-subjects factor of group (unsupported, looker and non-looker) and heading at test (forward-facing, else). Mean performance of each group (shown in Fig. 5) reveals a clear pattern.

There was a main effect of heading at test, with participants performing more accurately on forward-facing trials, *F*(1,62) = 38.87, *p* < 0.001, *η*_*P*_^*2*^ = 0.39. There was also a main effect of group, *F*(2,62) = 12.83, *p* < 0.001, *η*_*P*_^*2*^ = 0.29. The two factors significantly interacted, *F*(2,62) = 4.71, *p* = 0.01, *η*_*P*_^*2*^ = 0.13. Simple follow-up comparisons confirmed that the non-lookers performed significantly less accurately than the other groups on the more difficult, non-forward-facing trials: versus lookers, *t*(36) = 3.41, *p* = 0.002, *d* = 1.11, *BF* = 20.95, or unsupported participants, *t*(44) = 3.92, *p* < 0.001, *d* = 1.17, *BF* = 83.37. The same pattern was evident in the forward-facing trials: versus lookers, *t*(36) = 3.62, *p* < 0.001, *d* = 1.17, *BF* = 33.62, or unsupported participants, *t*(44) = 3.97, *p* < 0.001, *d* = 1.19, *BF* = 94.50. The comparisons between the lookers and unsupported participants were not significant irrespective of heading: forward-facing, *t*(44) = 0.23, *p* = 0.82, *d* = 0.7, *BF* = 0.30, and non-forward-facing, *t*(44) = 0.01, *p* = 1.00, *d* = 0.00, *BF* = 0.30. In summary, there was evidence of over-reliance on the overlay amongst participants in the supported group who looked less at the time-bearing plot (those with a low “look forward” ratio) as indicated by poorer accuracy as compared with both those supported participants who looked at the time-bearing plot more during training, and those who were not given access to the overlay.

A final question concerned who benefits from provision of the conformal display. Perhaps those with an inherent capacity for spatial reasoning are those who look forward most when it is displayed, and thus benefit from its presence. Put another way, perhaps the mental rotation test and our gaze measure during training are just two indicators of the same people; they do not capture unique variance. To test whether these predictors each make a unique contribution to explaining test performance of the supported group, we linearly regressed test accuracy on the non-forward-facing trials on both mental rotation scores and the tendency to look forward during training (on the non-forward-facing trials). The model explained a significant portion of variance in test accuracy, *F*(2,33) = 4.94, *p* = 0.01, and there was little indication of collinearity amongst the predictors, *VIF* = 1.02. In this model, mental rotation score, *b* = 0.37, *t* = 2.18, *p* = 0.04, significantly and positively associated with test accuracy performance (both coefficients reported in standardized form). Look forward ratio during training was not significant, *b* = 0.30, *t* = 1.94, *p* = 0.06, once mental rotation was accounted for. When the same regression was used to test for predictors of test performance on forward-facing trials, but predictors were individually significant (with no evidence of collinearity, *VIF* = 1.02): mental rotation score, *b* = 0.35, *t* = 2.43, *p* = 0.02, and look forward ratio, *b* = 0.39, *t* = 2.68, *p* = 0.01.

## Discussion

We provide clear evidence for the efficacy of a VR visual overlay in supporting the interpretation of time-bearing plots by novice adults. The overlay took the allocentric information in a traditional time-bearing plot, and mapped it into an aligned, semi-transparent, conformal display that participants could use in addition to the traditional display. Judgments were between 11° (easier trials) and 20° (harder trials) more accurate in participants given the overlay than those without. The presence of the overlay also resulted in faster judgements and appeared to require less time inspecting the time-bearing plot (lower “look forward” gaze ratios).

Unfortunately, there did not appear to be any positive transfer to an unassisted test phase. The supported group markedly underperformed at test. That is, they had a steeper decline in response speed and accuracy from training to test than the unsupported group, and were significantly less accurate on test trials than the unsupported group. On the most difficult trials (the non-forward-facing trials), this represented a reversal of a 20° advantage for the supported group to a 14° advantage for the unsupported group. There were no gross changes in orienting (look forward and look target ratios) and response speed between groups in the final test phase. Instead, the primary difference appeared to lie in lowered spatial judgment accuracy in people who were previously exposed to the overlay.

### Spatial skills and task difficulty

The impacts of our overlay manipulation were more evident on more difficult trials. Averaged across all groups and phases, people performed significantly less accurately and were significantly slower on trials in which the target vessel was moving (8° and 1.3 s) or when their ownship’s heading misaligned with the time-bearing plot (11° and 0.5 s). We will consider each manipulation in isolation. First, when the target vessel was moving, participants were forced to interpret a time-bearing plot in which the contact was depicted as a curved line, with non-linear horizontal motion. Participants needed to assess where the vessel was now (by interpreting the upper most data point in the display) and where it was 20 s ago (by interpreting where the signal crossed the 20 s gridline). By contrast, on static trials, there was a single almost vertical trace.

Viewed from the perspective of spatial cognition, the static trials could be considered an extrinsic-static task. They required participants to perform a relatively elementary perspective shift to map the Cartesian plot (time by bearing) with one’s own egocentric perspective (e.g. “up” = “in front”). This translation is slightly more complicated for targets behind the viewer, as “behind” is mapped as “far left” or “far right,” because the horizontal axis represents rotation (yaw), not distance. By contrast, the dynamic trials were an extrinsic-dynamic task, and loaded more heavily on mental rotation skills. Participants could retain their perspective and spatial framework, but mapping the target’s horizontal motion on the time-bearing plot to the participants’ own egocentric spatial coordinates meant performing at least two non-linear transformations (rotations about the viewer’s vertical axis). This was a more complex judgment which was made more slowly and with more error.

However, the largest impact on people’s performance was their own heading. Ownship heading was randomly chosen per trial and represented as a dotted blue line on the time-bearing plot (and equivalently on the overlay). This manipulation significantly interacted with phase and group, such that the costs and benefits of the overlay were most evident on trials in which the participants’ orientation did not match that of the time-bearing display. While the heading misalignment did not impact on the broad, skill-based classification of our task (it remains extrinsic-dynamic or extrinsic-static, depending upon boat speed), it did alter the cognitive requirements of the participant. When heading was markedly misaligned with the plot, participants needed to first shift their perspective to that of the dotted blue line, before performing the transformations discussed above (Fig. 2 shows an example of the kind of transformation required). This initial shift in perspective was only a translation (which should be easier than a rotation, Michelon & Zacks, 2006). However, in combination with the requirement to read the Cartesian plot as polar, it effectively constitutes a perspective rotation too.

Importantly, these two aspects of the task are likely intersectional. We treated these aspects separately, partly to reflect pre-existing distinctions in the spatial cognition literature (Uttal et al., 2013; Hegatry & Waller, 2004) and partly to exert statistical control and retain power. Yet anecdotally it was trials in which the two factors needed to be interpreted in conjunction that were reported as most difficult for participants. This was further complicated if, during a dynamic trial, the horizontal motion crossed either one’s own heading, or the left/right extreme of the plot thereby wrapping around onto the other side of the plot.

Such discontinuities remain a persistent challenge whenever a 360-degree camera must be mapped to a set of panels. Bell et al (2025) recently studied how 360-degree visuals from a periscope are best represented in a series of displays and found that user alignment (similar to heading in the present task) appeared to impact performance more than the introduced discontinuities across panels (see also Michailovs et al., 2022, 2024). Yet this was a static task, in which the targets did not move laterally across discontinuities. The optimal visual arrangement under more dynamic conditions is not yet known. It is possible that conformal VR/AR displays that do not have such discontinuities (at least laterally) may offer further improvements beyond existing display formats. Unfortunately, our ability to quantify the impact of the intersection of all these factors was challenged by the fact that our trials randomized ownship heading and target vessel velocity, so people experienced different quantities of these more challenging trials. Future work focused on exploring the intersection of these spatial skill requirements, and people’s ability to navigate display discontinuities, could carefully plan and control headings on a trial-by-trial fashion to directly address these questions.

In quantifying pre-existing spatial abilities, we used a simple pen-and-paper test of mental rotation skill. Our empirical focus was on the design, implementation and efficacy of the novel visual aid, so we sought a simple, heuristic measure of pre-existing abilities to constrain any differences amongst our participants that were so large as to obscure our measurement of the efficacy of the overlay. This simple approach proved fruitful in that it allowed us to test the efficacy of random-assignment by examining pre-existing differences between our participant groups, and afforded a simple manipulation check of our time-bearing interpretation task; if the task taxes spatial abilities generally, then a simple test of spatial abilities should predict performance (as it did). Indeed, there is evidence that distinct spatial abilities tend to correlate strongly with each other (Hegarty & Waller, 2004). However, a more nuanced approach to quantifying spatial capacities would likely have been more informative. Different aspects of our task conceptually load on differentiable spatial skills (Kozhenikov & Hegarty, 2001), yet we were unable to examine these narrow hypotheses due to our simplistic assessment of pre-existing capacity. Future research should use a standardized task to assess perspective-taking skills (such as the computerized spatial orientation test; Friedman et al., 2020) and be mindful that people vary not only in spatial skill but in preferred spatial format (Pazzaglia & De Beni, 2001). Until these distinctions are examined, it remains an open question as to who would benefit the most from our overlay.

### Evaluation of the overlay

In aggregate, the visual overlay was helpful. It reduced the perspective-taking cognitive load on the participant by translating the time-bearing plot so as to align with user perspective. Similarly, it performed the rotation computations to horizontally align bearing information to reflect the user’s egocentric perspective. In essence, it off-boarded cognitively effortful aspects of spatially interpreting the time-bearing plot, thereby increasing judgment accuracy and reducing response latency.

Perhaps the most stringent test of the efficacy of the overlay lies in whether it alters the pattern of who performs well, and which trials are difficult. Suppose there were two tasks: lift 100 kg or lift 10 kg. In the absence of any support, one task would be much more difficult than the other, and performance on both tasks could be predicted by a pre-existing ability (strength). However, if we gave participants access to a forklift, then both relationships would break; both weights are equally effortless to lift, and the relationship between strength and lifting capacity would be removed. In applying the same criteria to our overlay, we see broadly the same pattern. The underlying relationship between a simple test of spatial ability (a pen-and-paper mental rotation test) and accuracy in pointing judgments was no longer obtained when the visual overlay was present. In other words, when the overlay was present, people’s ability to perform accurate pointing judgments appeared to no longer depend on their pre-existing spatial skills. The provision of the overlay also significantly reduced the differences in response accuracy between difficult and easy trials when it was present. To confirm this, we performed a simple t test comparing the performance of the supported group during the training phase on the forward-facing and non-forward-facing trials: it was not significant, with anecdotal evidence for equivalence, *t*(37) = 1.50, *p* = 0.14, *d* = 0.24, *BF* = 0.49. The overlay clearly changed how people performed the task.

Unfortunately, our overlay acted more like a crutch than a scaffold (at least in aggregate). Our design intention was that by offboarding some of the mental transformations needed to interpret the time-bearing plot, people would have cognitive resources available to learn the mapping between a Cartesian representation of bearing information (an x coordinate) and egocentric bearing (a person-centred outwards direction). Indeed, only when the overlay was present was the correct direction visually indicated concurrently with the console during the decision period. This temporal contingency is known to facilitate the fundamental mental associations often considered to underlie higher-order cognition, including spatial reasoning (Boakes & Costa, 2014). Returning to the weightlifting metaphor, we sought to offer something akin to an assisted pull-up machine; instead of people being faced with the requirement to lift a large weight in one go, we sought to take some portion of that weight so that people could use available resources to develop form and thus build capacity.

We were unsuccessful in this goal, as our data are more consistent with automation over-reliance and misuse (Parasuraman & Riley., 1997). Our overlay support was described and experienced as perfectly accurate and reliable, and the task it was built to support is complex. These values were chosen to maximize the difference between supported and unsupported conditions, but they are also important determinants of high levels of reliance (Lee & See, 2004). The sudden removal of the overlay was designed to test transfer and thereby assess the degree to which the support had scaffolded learning (Schmidt & Bjork, 1992). Yet the repeated presentation of a highly reliable, helpful overlay, in the face of an otherwise challenging task, likely self-reinforced the participants’ dependence on that overlay (inducing “reliance inertia”, Lee & Moray, 1994). Had our overlay continued to remain available, this high level of reliance could have constituted a high, but appropriate, level of reliance. While we did not declare that we would remove the overlay, we did repeatedly ask participants to use the overlay to *learn* how to interpret the time-bearing plots. Thus, the choice to completely rely on the overlay could be construed as a form of automation-induced complacency (Prinzel et al., 2001, 2005) whereby an operator partly or wholly cognitively disengages with the task at hand. Quantifying variability in susceptibility to this phenomenon across individuals is well-studied (Merritt et al., 2019; Parasuraman & Manzey, 2010; Prinzel et al., 2001, 2005).

By examining gaze patterns during training, we identified those participants who engaged most or least with the time-bearing plot during training (via their look-forward ratio), and we found that the participants who inspected the time-bearing plot more often showed better transfer to test. Indeed, a further analysis revealed that those participants in the supported group who maintained frequent gaze upon the time-bearing plot while the aid was present, performed just as well as the unsupported group at test when the support was removed. That is, they showed no evidence of reliance or induced complacency, while nevertheless benefiting from the aid when it was present.

This pattern of over-reliance being evident in only a subset of participants calls to mind the conclusion of Chen et al (2017), in one of the closest related studies to the present task. In their task, participants were tasked with interpreting a time-bearing “waterfall” plot, similar to the present study. However, in Chen et al’s (2017) task, participants were required to make a broad range of judgments about those contacts, to report on mental workload and to maintain awareness of several contacts presented simultaneously in a richly detailed, 30-min submarine simulation. Some participants were provided with automated assistance lines to help identify the behaviours of contacts (or “tracks”) that may be indicative of a potential adversary. In their study, there was evidence of their support tool leading to impaired performance on a standardized measure of situational awareness (Endsley, 1995), indicative of complacency, while simultaneously measuring benefits in other task-relevant judgments (closest point of approach, classification judgments). These findings were further complicated by differences observed when the task was implemented as a between-participants or a within-participants experiment (for example, closest point of approach judgments were not always superior with support). Clearly the calculation of costs and benefits of automation and decision supports in the performance of complex, multifaceted duties is a nuanced assessment. The present data contribute to this discussion by highlighting the value of granular behavioural data (gaze position) in identifying who will benefit from a support in a resilient manner, and whose performance gains are more brittle.

## Limitations

While the present work was informed by real operational duties, it favoured internal validity over external validity. The transition from cognition lab to the cockpit is challenging, and unlikely to be achieved in a single study (see Clarke et al., 2012 for a discussion). We sought tight experimental control at the cost of realism, and so the present experiment falls far short of an in situ test of a novel decision support prototype. Our participants were novices, and spatial skill can be markedly impacted by many forms of expertise (Calabrese & Marucci, 2006; Fernandez et al., 2011; Spence & Feng, 2010). The substantial, prolonged training schedules of professional submariners is likely to impact on that skill.

While we were unable to assess submariners directly in this project due to resourcing constraints, we did look at another source of expertise relevant to spatial and navigational skill: experience with video games. Experience with video games is associated with superior spatial and navigational performance, particularly those that require three-dimensional navigation as a core game element (Richardson & Collaer, 2011; Richardson et al., 2011; Yavuz et al., 2024). Surprisingly, regular gamers were no more likely to demonstrate the basic level of spatial competency required to engage in our task than were non-gamers (see *Determining Learners*). A second post hoc analysis examined whether more narrow experience with games that centre navigational requirements (such as first-person shooter, FPS, games) impacted initial performance, thereby indicating a pre-existing benefit for gamers. To do so, we further separated the included participants by those who noted FPS titles as their preferred games (*n* = 23) and compared them against everyone else (*n* = 42), and examined accuracy during the baseline phase. No significant differences was observed, *t*(63) = 0.18, *p* = 0.86, again suggesting that extensive experience with virtual spatial navigation had no detectable performance impact on the present task.

This does not, however, imply that an experience submariner would perform at the same level as our novice participants. The present task constitutes far transfer from all of the games participants listed experience with, but is fundamental to the complex spatial assessments routinely made to submariners. We therefore suspect that trained submariners would not show the same level of difficulty with our simplified task designed primarily for novices. However, realistic undersea information environments are more complex than we were able to model in novice learners, and so arguably present more opportunities for reduction in extraneous load associated with spatial transformations that could be offloaded or merged though user-interface innovation (Fay et al., 2019). So the question of benefit afforded by conformal display formats and information overlays more generally remains open. Future research should consider the role of separable forms of spatial cognition in these assessments, and the role of genuine expertise in performing and managing this cognitive load.

Finally, our decision to limit analysis to that of participants who demonstrated above-chance performance during training introduced an alternative explanation of the data. This decision was essentially a pragmatic one; we could only investigate people’s learning or performance when we were confident that they were not merely guessing, and we had no such confidence in those people who were consistently performing at chance levels. Another common approach to this issue of individual differences is to continue training participants until everyone reaches threshold performance. Yet this too induces imbalance because people then have differential levels of training (and task familiarity and fatigue). Nevertheless, our design choices may have meant that the presence of the overlay lifted some participants in the supported group to above chance responding who counterfactually would not have attained that level of performance had they instead been assigned to the unsupported group. More simply, we may have accidentally selected for pre-existing spatial ability in the unsupported group, but not in the supported group. If so, then it would follow that we would see greater performance in the unsupported group during the final test, in which support and feedback was withheld. Such test differences might therefore be considered a product of selection, not over-reliance: Those who could only lift the weight with a forklift were retained in one group, but not in the other.

This account is unlikely to be a sufficient explanation of the present findings. If we compare pre-existing mental rotation ability in the supported and unsupported group, there is no indication of a systematic difference between groups, *t*(61) = 0.70, *p* = 0.94, *d* = 0.02. Second, we used linear mixed models that included individual intercepts in our primary analyses, thereby controlling for some variance attributable to individual difference in initial skill levels. Finally, we directly examined performance in the initial baseline phase between the supported and unsupported groups. This comparison was not significant, but equally it would be premature to interpret this as compelling evidence of equivalence between the groups. Clarifying Bayesian analyses reveal no notable evidence either for or against the proposition that they were the same or different during the baseline phase. This ambiguous, weak comparison stands in stark contrast to the larger, more robust significant differences seen in the other two phases, which suggests that pre-existing differences alone cannot account for the much larger differences that followed. In summary, we see this incidental selectivity as an incomplete and insufficient explanation of the impact of the overlay on training or test.

## Conclusion

Novice participants performed localization judgments using simplified time-bearing plots of the same format used by submariners. This occurred in a VR simulation in which we provided an additional egocentric, conformal overlay for half of the participants. Providing this additional overlay improved the speed and accuracy of localization judgments. When that support was withdrawn, there was indication of over-reliance on the conformal display, in that formerly supported participants performed worse than formerly unsupported participants. This pattern varied across individuals. Poor performance at test was predicted by the way participants engaged with the aid, and by pre-existing spatial reasoning skill (mental rotation). Such observations may prove useful in training and selecting submariners, and in developing metrics to support the safe (and not over-reliant) future use of augmented reality supports for operational judgments.

## Data Availability

Data are currently available upon request as our initial ethics application did not explicitly include permission for public access. Other materials are available at: https://osf.io/jxptu/

## Abbreviation

SONAR: SOund Navigation And Ranging
